# Donation or Advertising? The Role of Market and Non-market Strategies in Corporate Legitimacy

**DOI:** 10.3389/fpsyg.2022.943484

**Published:** 2022-06-13

**Authors:** Ying Liu, Wei Liu, Yingbo Xu

**Affiliations:** Business School, Qingdao University, Qingdao, China

**Keywords:** marketing, non-market strategy, media coverage, advertising, donation, financial performance, big data, emotion

## Abstract

Although existing research has discussed the impact of market strategy or non-market strategy on corporate legitimacy thoroughly, there is limited research on the joint role of the two strategies. Based on the big data analysis of media coverage, this study addresses this research gap by using a sample of Chinese listed firms during 1999–2018. Our finding reveals that positive media coverage promotes corporate financial performance, and advertising intensity and corporate donation strengthen this relationship. However, the simultaneous application of market and non-market strategies diminishes the effect of both strategies on the expansion of corporate legitimacy. This study extends the literature on the impact of corporate strategies on corporate legitimacy by highlighting the joint role of the corporate market and non-market strategies.

## Introduction

Media coverage has recently received a great deal of attention from scholars who have found that a firm’s share price and valuation are heavily influenced by the media on various aspects of the firm. For instance, Dong et al. (2022) found that a limited amount of negative news may positively affect corporate financial performance by increasing awareness of a firm. Media coverage has different emotional tendencies, and when stakeholders receive positive emotional tendencies from news reports, they will have a better impression of the firm. In other words, media coverage is an important signal for firms, and stakeholders use it to form their impressions of the firm (Guldiken et al., 2017). Kölbel et al. (2017) found that media coverage of corporate social irresponsibility increases the likelihood of stakeholder sanctions by drawing attention to firms, and therefore firms may be exposed to higher financial risk. Media coverage also has a monitoring and regulatory role (Zhang and Chen, 2019), which is an important measure for stakeholders to evaluate the legitimacy of a firm (Young-Chul and Tai-Young, 2019).

However, the extent to which media coverage affects corporate financial performance can be influenced by different conditions. In fact, there are many previous studies on how corporate market and non-market strategies affect financial performance, respectively. The impact of corporate marketing strategies on financial performance has been widely studied (Zhang et al., 2020). When the corporate advertising intensity is higher (Nikolaeva and Bicho, 2010), key stakeholders (e.g., customers) can be aware of the positive activities done by the firms (Servaes and Tamayo, 2013). These activities can influence stakeholders’ evaluation of the firms (Vogler and Eisenegger, 2020), resulting in better financial performance.

When firms actively engage in donation activities, they will have a positive image, enhance customers satisfaction (Ali et al., 2019), improve relationships with stakeholders (Hou, 2019), and boost investors confidence (Babajee et al., 2021), which can lead to better corporate financial performance. Corporate social responsibility (CSR) and corporate political activities constitute the main elements of non-market strategies. Mellahi et al. (2016) studied the impact of CSR and corporate political activities on organizational performance, and they suggest that firms need to make adaptive non-market strategies when entering new markets in order to gain legitimacy in the face of local social pressures and priorities.

Therefore, we know from the existing literature that when faced with media coverage, firms make market and non-market strategies separately, which can have a positive impact on corporate financial performance. However, previous studies do not tell us a clear logic of how market and non-market strategies affect corporate financial performance when they are made simultaneously, and whether they are co-promotional or substitution.

To try to answer this question, we explore the role of two marginal conditions, corporate advertising intensity and corporate donations, from the perspective of corporate legitimacy and signaling theory, under the premise that media coverage can positively affect corporate financial performance. First, we argue that when media coverage of firms is a positive signal, it will attract stakeholders’ attention to firms, which in turn helps firms gain external legitimacy. Second, when firms increase their advertising intensity and donations, respectively, they will similarly have a positive impact on corporate financial performance. Finally, we also argue that market and non-market strategies, when pursued simultaneously, have a mutually substitutable binding effect on firms.

We use the sample of Chinese listed firms during 1999–2018 to conduct empirical tests, which attempts to make the following contributions. First, we explore the mechanisms of media coverage on corporate financial performance, the marginal effects of advertising intensity and donation from the perspective of external legitimacy, respectively. Second, we explore the impact on corporate financial performance when their market and non-market strategies occur simultaneously from the perspective of corporate strategic motives, which adds to our understanding of corporate strategic motives and has important practical implications.

## Hypotheses Developments

### Pursuit of Legitimacy *via* Media Coverage

Media coverage is an important factor that affects corporate financial performance. Media coverage regarding firm information can reduce the information asymmetry between internal and external stakeholders of the firm (Fang and Peress, 2009). It facilitates the communication between firms and the public while guiding public opinion (Dong et al., 2022). Simultaneously, media coverage provides market participants with information about the legitimacy of a firm (Young-Chul and Tai-Young, 2019). As a signal, positive media coverage of firm news draws the attention of stakeholders (e.g., government, public), who tend to judge the firm positively. It can also reduce information asymmetry about their legitimacy (Mumi et al., 2018) and helps firms to gain external legitimacy and improve financial performance. Therefore, we propose the following hypothesis:

**H1:** Media coverage will have a positive impact on corporate financial performance.

### The Moderating Role of Advertising Intensity

Some studies have shown that a positive firm image increases customer satisfaction and loyalty, and hence it can lead to better performance by increasing the price of products (Ali et al., 2019). Firms aim to formulate market strategies because it can enhance corporate image (Kim et al., 2011). As a type of market strategy, advertising intensity is an important factor of firm reputation (Wang and Qian, 2011). Firms use it as a differentiation market strategy (Zhang et al., 2009) and a means to acquire legitimacy (Özturan and Grinstein, 2021). When stakeholders recognize differentiation, they learn more about the firms, including their products and practices (Ganda and Milondzo, 2018). While firms build credibility and enhances their image, external stakeholders can make a good assessment of corporate legitimacy, which generates resources in turn (e.g., financial investments, talented employees, new competencies) (Nikolaeva and Bicho, 2010). Therefore, an increasing number of stakeholders will pay attention to media coverage of firms with higher advertising intensity. As a signal, an advertisement of firm provides a positive message about the firm, which can increase customer loyalty and improve corporate reputation and image (Taoketao et al., 2018). When positive news about the firms are published, the attention of stakeholders helps firms acquire external legitimacy, which enhances their financial performance. In contrast, when advertising intensity is lower than before, stakeholders will ignore more positive news about the firms because they cannot focus on corporate messages. Therefore, we propose the following hypothesis:

**H2:** Advertising intensity enhances the positive relationship between media coverage and corporate financial performance.

### The Moderating Role of Donation

Corporate social responsibility is an important branch of non-market strategies (Mellahi et al., 2016), which is a source of external legitimacy for firms and managers. In an altruistic society, there is more positive media coverage and more positive comments about firms, stakeholders (e.g., the public, employees, investors, local communities, governments) will pay more attention to the performance of firms in all dimensions, and they also expect firms to take social responsibility (Peloza and Shang, 2010). At the institutional level, firms gain a broader external legitimacy by undertaking CSR. In other words, they fulfill unwritten rules of the “social contract” that the organization must abide by Scott (1987). Donations also imply that firms are willing to allocate resources to stakeholders (Barnett, 2007). Firms will acquire more critical resources from stakeholders after them receive the signal (Su et al., 2014). For instance, during the flooding event that occurred in Henan Province, China in 2021, the Chinese firm Hongxing Erke donated 50 million RMB to the disaster area. It acquired public supports and increased its sales 52 times after donating, with total sales of 150 million RMB. By donating when they are not hit by negative news, firms gain t trust from stakeholders, improve their credibility (Kim, 2019), help themselves to gain external legitimacy and enhance their financial performance. Therefore, we propose the following hypothesis:

**H3:** Corporate donation enhances the positive relationship between media coverage and corporate financial performance.

### The Interaction Between Advertising Intensity and Donation

Similarly in 2021, during the flooding event in Henan Province, Chinese firm Pinduoduo announced a donation of 100 million RMB to help Henan Province. Their technical team developed an “emergency relief material feedback app” quickly, collecting disaster relief needs from affected areas and providing information matching and material support. However, contrasting with the public positive assessment of Hongxing Erke, after positive media coverage of this charitable activity, the public perceived Pinduoduo’s donation as hypocritical to offset the negative impact of the previous group scandal. This leads us to ask the question: Why did the same positive media coverage lead to different evaluations by stakeholders?

To some extent, the non-market strategy of corporate donation is altruistic. In other words, in an altruistic activity, the actor decides to voluntarily give an object to the recipient without expecting a direct return (Barman, 2017). In order to be socially responsible by fulfilling the “social contract,” firms usually make non-market strategic decisions (Scott, 1987), rather than simply for the benefit of firms. This is a positive signal from the firm to the stakeholders (Connelly et al., 2011). Then, when stakeholders provoke activities done by firms out of altruism, they can, in turn, amplify positive comments about firms in media coverage, which will help firms gain more external legitimacy. However, advertising is characterized differently from donations in that it is essentially self-serving. Firms want to expand their visibility and gain good reputation through advertising, with the ultimate goal of improving financial performance. When a firm makes decisions out of financial interest, signals become ambiguous because the information of organization conveyed is no longer automatically interpreted by stakeholders as positive, it can be interpreted positively or negatively by stakeholders (Ogunfowora et al., 2016). However, some researches have shown that firms make decisions in an altruistic position, stakeholders are able to perceive the corporate intentions (Aguilera et al., 2007) and change their evaluation of the organization (Skarmeas and Leonidou, 2013). Therefore, when market and non-market strategies collide, they tend to trigger suspicion and perceptions of corporate hypocrisy among stakeholders (Du and Bhattacharya, 2010). Thus, we can explain why the same positive media coverage can lead to different stakeholder evaluations, and we propose the following hypothesis:

**H4:** The positive relationship between media coverage and corporate financial performance is weakened when corporate marketing and corporate giving occur together.

## Methodology

### Sample and Measures

We used a sample of Chinese listed firms during 1999–2018 to conduct empirical tests, with the China Stock Market Accounting Research (CSMAR) database as the main data source at the firm level. The data starts in 1999 to avoid the anomalous impact of the 1998 Asian financial crisis on Chinese listed firms and ends in 2018 to avoid the anomalous impact of the COVID-19 pandemic in 2019.

#### Dependent Variable

We used one-year-lagged *Tobin’s Q* to measure corporate financial performance, which is the ratio of the market value of capital to the cost of reproduction. As a long-term indicator of the market value of a firm (Magomedova, 2016), Tobin’s Q reflects to some extent the future profitability of the firm (Vithala et al., 2004), and it is also frequently used as a measure of financial performance (e.g., Surroca et al., 2010; Servaes and Tamayo, 2013; Liu et al., 2021).

#### Independent Variables

We used the natural logarithm of media emotional scores on corporate reports as the independent variable, *Media coverage*. We first mined all the news content of paper and Internet medias about the firms based on the Wisers News Database through the technique of big data. After excluding news with low credibility and importance and cleaning the content, we followed previous studies and used a machine learning model to discriminate emotional tendencies based on the positive part of the content (Piotroski et al., 2017). In addition, we assigned different weights to each paragraphs, and finally obtained a comprehensive positive score of media coverage of each firm.

We measured one moderating variable, *Donation*, as the natural logarithm of the amount of a firm’s charitable giving in a year. We measured another moderating variable, *Advertising intensity*, as the natural logarithm of the amount of advertising marketing costs a firm spending over the course of a year.

#### Control Variables

We considered two levels of control variables. First, at the firm level, we included Firm size, *Firm age*, *Debt ratio*, *Cash flow*, *State ownership* to consider their potential impact on corporate financial performance. Second, at the regional level, we included *Province GDP growth* and *Province media*. There are disparities in economic development among different provinces in East, Central and West China, so the level of media development in different provinces also has an impact on the quantity and quality of media coverage. We measured *Province GDP growth* in terms of GDP growth in each province relative to last year, and *Province media* in terms of the total number of employees in the media industry in one province. Finally, we also included *Industry dummies* and *Province dummies*.

### Estimation Method

Our sample data is panel structured covering years from 1999 to 2018 and the dependent variable is a continuous variable. After completing the Hausman test, we used multidimensional panel fixed effects model, estimated by least squares.

## Results

### Descriptive Statistics and Correlation

The descriptive statistics and correlation matrix of the main variables are shown in Table 1.

**TABLE 1:** Descriptive statistics and correlation matrix.

Variables	Mean	S.D.	(1)	(2)	(3)	(4)	(5)	(6)	(7)	(8)	(9)	(10)	(11)
(1) Tobin’Q	2.436	2.186	1.000										
(2) Firm size	22.098	1.449	−0.579	1.000									
(3) Firm age	16.069	5.431	−0.068	0.138	1.000								
(4) Debt ratio	0.442	0.630	−0.129	0.136	0.086	1.000							
(5) State ownership	0.039	0.124	−0.100	0.163	0.048	0.026	1.000						
(6) Cash flow	10.363	15.986	−0.032	0.123	−0.027	−0.060	0.026	1.000					
(7) Province GDP growth	0.084	0.017	−0.051	−0.092	−0.106	0.024	0.055	−0.030	1.000				
(8) Province media	21.737	17.407	0.048	0.104	−0.025	−0.034	−0.016	0.011	−0.370	1.000			
(9) Advertising intensity	17.462	3.713	−0.099	0.043	−0.065	−0.036	−0.072	0.079	−0.045	0.019	1.000		
(10) Donation	8.41	6.079	−0.199	0.262	−0.018	0.004	−0.003	0.089	0.054	−0.015	0.184	1.000	
(11) Media coverage	3.033	1.999	−0.130	0.403	−0.019	−0.031	0.066	0.112	−0.016	0.150	0.052	0.173	1.000

*N = 12,620; correlations greater than |0.02| are significant at the 0.05 level.*

### Hypotheses Testing

Table 2 reports the baseline estimation results. Hypothesis 1 predicts a positive relationship between media coverage and corporate financial performance. As shown in Model 1, *Media coverage* is positive and significant at the 0.1% level (β = 0.108, *p* < 0.001). It shows that when the media coverage score is higher, the corporate financial performance is better, validating H1. Hypotheses 2 and 3 predict the strengthening moderating effects. As shown in Model 2, *Media coverage* × *Advertising intensity* is positive and significant at the 0.1% level (β = 0.022, *p* < 0.001). As shown in Model 3, *Media coverage* × *Donation* is positive and significant at the 0.1% level (β = 0.012, *p* < 0.001). Therefore, H2 and H3 were confirmed. H4 predicts that the positive relationship between media coverage and corporate financial performance is weakened when market and non-market strategies occur simultaneously. As shown in Model 4, *Media coverage* × *Advertising intensity* × *Donation* is positive and significant at the 0.1% level (β = −0.001, *p* < 0.001), thus H4 is strongly supported.

**TABLE 2:** Estimates for corporate financial performance.

Variables	DV: Tobin’ Q				Variables	DV: Tobin’ Q			
	Model 1	Model 2	Model 3	Model 4		Model 5	Model 6	Model 7	Model 8
Firm size	−0.934***	−0.938***	−0.951***	−0.938***	Firm size	−0.894***	−0.901***	−0.908***	−0.901***
	(0.015)	(0.015)	(0.014)	(0.014)		(0.017)	(0.017)	(0.017)	(0.017)
Firm age	0.006	0.004	0.004	0.003	Firm age	0.007*	0.006	0.006*	0.006*
	(0.003)	(0.003)	(0.003)	(0.003)		(0.003)	(0.003)	(0.003)	(0.003)
Debt ratio	−0.102***	−0.129***	−0.125***	−0.141***	Debt ratio	−1.366***	−1.376***	−1.366***	−1.384***
	(0.024)	(0.024)	(0.024)	(0.024)		(0.088)	(0.088)	(0.088)	(0.088)
State ownership	0.007	0.066	0.053	0.054	State ownership	0.054	0.088	0.092	0.086
	(0.126)	(0.125)	(0.125)	(0.124)		(0.121)	(0.121)	(0.121)	(0.121)
Cash flow	0.005***	0.005***	0.005***	0.005***	Cash flow	0.004***	0.004***	0.004***	0.004***
	(0.001)	(0.001)	(0.001)	(0.001)		(0.001)	(0.001)	(0.001)	(0.001)
Province GDP growth	−16.325***	−17.199***	−16.897***	−17.195***	Province GDP growth	−18.736***	−19.061***	−18.973***	−18.967***
	(1.262)	(1.252)	(1.252)	(1.248)		(1.291)	(1.287)	(1.287)	(1.287)
Province media	0.017***	0.017***	0.018***	0.018***	Province media	0.017***	0.017***	0.017***	0.017***
	(0.003)	(0.002)	(0.003)	(0.002)		(0.002)	(0.002)	(0.002)	(0.002)
Advertising intensity	−0.041***	−0.041***	−0.032***	−0.033***	Advertising intensity	−0.030***	−0.032***	−0.027***	−0.028***
	(0.006)	(0.006)	(0.006)	(0.006)		(0.006)	(0.006)	(0.006)	(0.006)
Donation	−0.015***	−0.016***	−0.020***	−0.019***	Donation	−0.017***	−0.018***	−0.020***	−0.020***
	(0.003)	(0.003)	(0.003)	(0.003)		(0.003)	(0.003)	(0.003)	(0.003)
Media coverage	0.108***	0.105***	0.105***	0.109***	Media coverage	0.400***	0.387***	0.385***	0.384***
	(0.009)	(0.009)	(0.009)	(0.009)		(0.015)	(0.015)	(0.015)	(0.016)
Media coverage × Advertising intensity		0.022***		0.015***	Media coverage × Advertising intensity		0.009**		0.007
		(0.002)		(0.002)			(0.003)		(0.003)
Media coverage × Donation			0.012***	0.009***	Media coverage × Donation			0.005***	0.006**
			(0.001)	(0.001)				(0.001)	(0.001)
Advertising intensity × Donation		0.005***	0.007***	0.007***	Advertising intensity × Donation		0.004***	0.007***	0.006***
		(0.001)	(0.001)	(0.001)			(0.001)	(0.002)	(0.002)
Media coverage × Advertising intensity × Donation				−0.001***	Media coverage × Advertising intensity × Donation				−0.001*
				(0.000)					(0.000)
Industry dummies	Yes	Yes	Yes	Yes	Industry dummies	Yes	Yes	Yes	Yes
Province dummies	Yes	Yes	Yes	Yes	Province dummies	Yes	Yes	Yes	Yes
Constant	24.499***	24.681***	24.767***	24.537***	Constant	23.108***	23.371***	23.416***	23.319***
	(0.324)	(0.322)	(0.322)	(0.324)		(0.355)	(0.355)	(0.355)	(0.357)
*F*-value	635.23***	560.39***	557.41***	487.72***	*F*-value	651.18***	551.62***	552.56***	474.72***
Adj R-squared	0.415	0.426	0.425	0.429	Adj R-squared	0.452	0.456	0.456	0.456
Observations	12,620	12,620	12,620	12,620	Observations	11,409	11,409	11,409	11,409

Standard errors are in parentheses; ***p < 0.001, **p < 0.01, *p < 0.05.

### Robustness Check

We also tested the robustness of the results by using the alternative measure of the independent variable, media coverage, which is measured as the emotional scores of total coverage (not the only positive part) of each firm. As shown in Model 5, *Media coverage* is positive and significant at the 0.1% level (β = 0.400, *p* < 0.001). In Models 6 and 7, *Media coverage* × *Advertising intensity* is positive and significant at the 1% level (β = 0.009, *p* < 0.01), and *Media coverage* × *Donation* is also positive and significant at the 0.1% level (β = 0.005, *p* < 0.001), thus supporting H2 and H3. In Model 8, *Media coverage* × *Advertising intensity* × *Donation* is positive and significant at the 5% level (β = −0.001, *p* < 0.05). All these results are similar to the main results in Models 1-4 and reveal that our results are robust.

## Discussion and Conclusion

We explore the impact of media coverage on corporate financial performance in this study and investigate the marginal conditional effects of corporate market and non-market strategies. Using the sample of Chinese listed firms during 1999–2018, we found that when media coverage is more positive, corporate financial performance is better. When a firm’s advertising intensity or donation is higher, the firm can gain more stakeholder attention and external legitimacy in perceiving a positive signal, which positively affects financial performance. Furthermore, we explore the impact of media coverage on corporate financial performance when market and non-market strategies occur simultaneously. We argue that firms have different motivations for making decisions about market and non-market strategies. Therefore, when strategies occur simultaneously, they interact and create a binding effect, which in turn reduces corporate financial performance.

The main contribution of this study is that we highlight the impact on firms when market and non-market strategies are pursued simultaneously. Previously, when it comes to corporate financial performance, most studies have focused on the impact of a single strategy (e.g., Wang and Qian, 2011; Babajee et al., 2021). Although some scholars have argued that market and non-market strategies can help firms achieve better financial performance when they are in balance (Al-Shammari et al., 2021), we do not know the specific underlying mechanisms of their effects. Our findings give a way forward for this part of the literature.

There are also some limitations in this study. First, we use data from Chinese listed firms and do not consider non-listed firms. Compared to listed firms, non-listed firms receive less media attention and may be more susceptible to factors such as local policies and managerial appointments. The motivations for adopting market and non-market strategies in the face of media coverage may differ from those of listed firms. As a result, it may have different impacts on corporate financial performance. Second, our research context is limited to China. Although China is very representative as an emerging economy, unlike developed Western capitalist countries, Chinese firms may be more advantageous in developing corporate market and non-market strategies. This is brought about by the long-standing Confucian culture and the Chinese people’s identification with ideas such as willingness to contribute, so our findings need to be further tested in other contexts.

## Data Availability Statement

The original contributions presented in this study are included in the article/supplementary material, further inquiries can be directed to the corresponding author.

## Author Contributions

YL drafted the manuscript. YL and YX collected, analyzed, and interpreted the data. WL designed the study and instructed the project. All authors contributed to the work and approved it for publication.

## Conflict of Interest

The authors declare that the research was conducted in the absence of any commercial or financial relationships that could be construed as a potential conflict of interest.

## Publisher’s Note

All claims expressed in this article are solely those of the authors and do not necessarily represent those of their affiliated organizations, or those of the publisher, the editors and the reviewers. Any product that may be evaluated in this article, or claim that may be made by its manufacturer, is not guaranteed or endorsed by the publisher.
